# Factors Associated With Certainty Regarding Medical Aid in Dying Laws in a National Sample of U.S. Adults

**DOI:** 10.1093/geroni/igaf122.867

**Published:** 2025-12-31

**Authors:** Elissa Kozlov, Samuel Nemeth, Elizabeth Luth

**Affiliations:** Rutgers School of Public Health, Piscataway, New Jersey, United States; Purdue University, West Lafayette, Indiana, United States; Rutgers University, New Brunswick, New Jersey, United States

## Abstract

Medical Aid in Dying (MAID) is a legal medical option for terminally ill patients in a growing number of U.S. states, covering over one-fifth of the population. Using a national convenience sample of 3,221 U.S. adults in states where MAID is legal (n = 2,159) and not legal (n = 1,062), this study examines predictors of certainty regarding MAID legality (correctly identifying legality, incorrectly identifying legality, or not knowing). Controlling for sociodemographic factors and attitudes toward MAID, lower socioeconomic status was significantly associated with lower odds of correctly identifying MAID legality nationwide. Compared to financially “comfortable” individuals, those with “enough” (AOR: 0.71, p < 0.001) or “not enough” (AOR: 0.61, p < 0.001) to make ends meet had lower odds of correctly stating MAID is legal anywhere in the U.S. Similarly, those with some college (AOR: 0.75, p < 0.01) or a high school education or less (AOR: 0.66, p < 0.001) were less likely to correctly identify MAID legality. Being non-Hispanic Black (AOR: 0.62, p < 0.05), having a high school education or less (AOR: 0.52, p < 0.001), and reporting financial insecurity (AOR: 0.63, p < 0.05) were associated with incorrectly stating MAID was illegal in their state. In states where MAID is legal, longer duration of legality was associated with greater awareness (AOR: 1.06, p < 0.001). These findings suggest that certainty about MAID laws varies by socioeconomic and demographic factors, which may have implications for informed consent and patient-centered end-of-life decision-making.

